# B-ALL Complexity: Is Targeted Therapy Still A Valuable Approach for Pediatric Patients?

**DOI:** 10.3390/cancers12123498

**Published:** 2020-11-24

**Authors:** Stefano Ratti, Annalisa Lonetti, Matilde Y. Follo, Francesca Paganelli, Alberto M. Martelli, Francesca Chiarini, Camilla Evangelisti

**Affiliations:** 1 Department of Biomedical and Neuromotor Sciences, University of Bologna, Via Irnerio 48, 40126 Bologna, Italy; stefano.ratti@unibo.it (S.R.); matilde.follo@unibo.it (M.Y.F.); francesca.paganell16@unibo.it (F.P.); alberto.martelli@unibo.it (A.M.M.); 2 Giorgio Prodi Cancer Research Center, S. Orsola-Malpighi Hospital, University of Bologna, Via Massarenti, 11, 40138 Bologna, Italy; annalisa.lonetti2@unibo.it; 3 CNR Institute of Molecular Genetics Luigi Luca Cavalli-Sforza, Via di Barbiano 1/10, 40136 Bologna, Italy; 4 IRCCS Istituto Ortopedico Rizzoli, Via Di Barbiano 1/10, 40136 Bologna, Italy

**Keywords:** childhood, B-ALL, signaling pathway, target therapy

## Abstract

**Simple Summary:**

B-ALL is the more frequent childhood malignancy. Even though significant improvements in patients’ survival, some pediatric B-ALL have still poor prognosis and novel strategies are needed. Recently, new genetic abnormalities and altered signaling pathways have been described, defining novel B-ALL subtypes.Innovative targeted therapeutic drugs may potentially show a great impact on the treatment of B-ALL subtypes, offering an important chance to block multiple signaling pathways and potentially improving the clinical management of B-ALL younger patients, especially for the new identified subtypes that lack efficient chemotherapeutic protocols. In this review, we shed light on the up-to-date knowledge of the novel childhood B-ALL subtypes and the altered signaling pathways that could become new druggable targets.

**Abstract:**

B-cell acute lymphoblastic leukemia (B-ALL) is a hematologic malignancy that arises from the clonal expansion of transformed B-cell precursors and predominately affects childhood. Even though significant progresses have been made in the treatment of B-ALL, pediatric patients’ outcome has to be furtherly increased and alternative targeted treatment strategies are required for younger patients. Over the last decade, novel approaches have been used to understand the genomic landscape and the complexity of the molecular biology of pediatric B-ALL, mainly next generation sequencing, offering important insights into new B-ALL subtypes, altered pathways, and therapeutic targets that may lead to improved risk stratification and treatments. Here, we will highlight the up-to-date knowledge of the novel B-ALL subtypes in childhood, with particular emphasis on altered signaling pathways. In addition, we will discuss the targeted therapies that showed promising results for the treatment of the different B-ALL subtypes.

## 1. Introduction

B-cell acute lymphoblastic leukemia (B-ALL) is a group of blood malignancies that results from the clonal expansion of transformed B-cell precursors, representing around 25% of all pediatric tumors and more than 80% of pediatric ALL [1].

B-ALL is characterized by recurrent cytogenetic and molecular aberrations, including non-random chromosomal rearrangements, aneuploidy, gene deletions and amplifications [2]. All these alterations may lead to leukemogenesis by interfering with multiple signaling pathways or modulating hematopoietic transcription factors, epigenetic modifiers, cytokine receptors and tyrosine kinases (TK).

B-ALL prognosis in children has considerably been improved over the last few years, with current survival rates exceeding 85%, largely due to intensification of standard chemotherapy and advances in risk classification, supportive care and minimal residual disease (MRD) monitoring, making this one of the main achievements of oncology [3].

The outcome of pediatric B-ALL depends on different factors, such as the elevated heterogeneity at both molecular and clinical levels. Despite this marked heterogeneity, all B-ALL patients are treated with the same chemotherapeutic protocol. Thus, precision medicine approaches intending to increase the cure rate and, at the same time, reduce side effects are needed for B-ALL patients.

Over the last decade, novel approaches have been used to understand the molecular bases of B-ALL, including next-generation sequencing techniques and integrated genomic analyses, identifying new genes and altered signaling pathways with important prognostic and therapeutic significance [4]. These recent findings may impact on B-ALL, by improving the clinical risk stratification and treatment.

**Figure 1:**
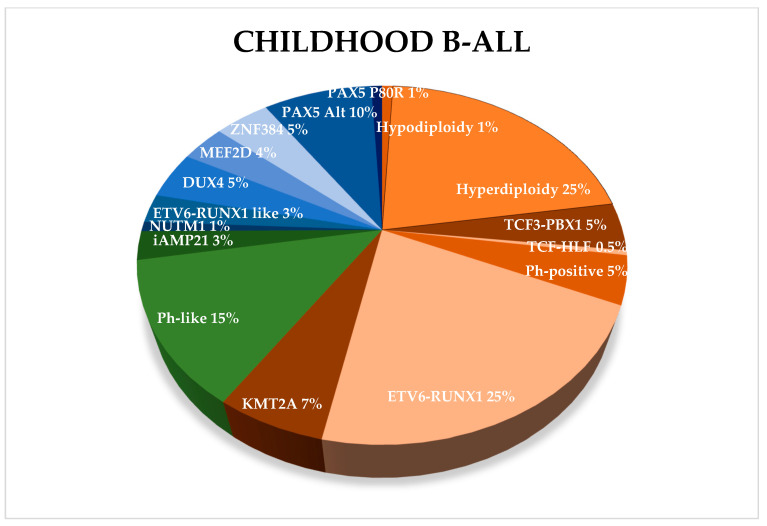
Frequency of childhood B-ALL subtypes. Established subtypes as defined by WHO in 2016 are indicated in orange, provisional entities in green, while new B-ALL subtypes in blue.

New genetic abnormalities, affecting the risk assessment and patients’ stratification, have been recently reported, defining novel B-ALL subtypes, characterized by frequent genetic alterations or chromosomal translocations. Some of them were included in the most recent World Health Organization (WHO) classification [5] (Figure 1).

In B-ALL, the aberrant proliferation of leukemic cells is correlated with deregulation of pathways and inhibition of different signaling networks, either alone or in association with conventional B-ALL drugs or other targeted therapies, could provide a rationale for the study of novel clinical protocols, particularly in the setting of chemo-resistance. In this scenario, the growing detailed studies of B-ALL biology have provided novel opportunities for the development of alternative and more effective therapeutic approaches that may lower the mortality rate and the systemic side effects caused by the chemotherapy currently used in B-ALL treatment.

Here, we will discuss the current state of knowledge of the genetic basis of new B-ALL subtypes, with a particular emphasis on altered signaling pathways.

## 2. Aneuploidy B-ALL

Aneuploidy is referred to the condition of an abnormal number of chromosomes and is one of the most common genetic alterations in pediatric B-ALL, identified several years ago. However, the recent and more precise characterization of B-ALL aneuploidy subtypes led to a better identification of risk stratification and treatment, since masked hypodiploidy could be confused with hyperdiploidy, causing a mistaken classification, a different outcome and, in turn, a treatment failure. Hypodiploidy represents a group of B-ALL with <44 chromosomes. Initially identified as a single subtype, it is actually divided into two groups that show distinct mutational profiles and are characterized by different transcriptional profiles and genetic variations: near-haploid ALL (<30 chromosomes) and low-hypodiploid ALL (31–39 chromosomes) [6]. Hyperdiploidy (>46 chromosomes) may be subdivided into low-hyperdiploidy (47–50 chromosomes) [7] and high-hyperdiploidy (51–56 chromosomes) [8].

### 2.1. Hypodiploidy

Near-haploid ALL arises in about <1% of pediatric B-ALL while low hypodiploidy occurs in 2% of cases. The prognosis of both low hypodiploid and near haploid is unfouvarable. Nevertheless, it has been recently demonstrated that low hypodiploid ALL treated within MRD-guided therapy and with good MRD clearance show a better outcome [9].

Near-haploid ALL is characterized by genetic alterations involving Ras signaling pathway, Receptor Tyrosine Kinase (RTK) signaling and IKZF3 [6], whereas *CDKN2A/B*, *IKZF2* and RB1 alterations or *TP53* germline mutations are frequently found in low-hypodiploid ALL [10].

A recent study showed that, in a B-ALL patient-derived xenograft (PDX) model, hypodiploid cells are sensitive to the B-cell lymphoma (Bcl) 2 inhibitor Venetoclax (ABT-199), suggesting that Bcl2 inhibition could be a promising strategy for the treatment of hypodiploid B-ALL [6,11].

Importantly, the efficacy of Venetoclax has been already established in chronic lymphocytic leukemia (CLL) and other blood malignancies, showing an acceptable safety profile [12], thus entering into regimens for the treatment of CLL patients [13]. Moreover, Bcl2 inhibitors entered in clinical trials for relapsed or refractory B-ALL patients (NCT03504644, NCT03808610, NCT03181126).

Hypodiploid B-ALL cells exhibit activation of Ras and phosphoinositide-3 kinase (PI3K)/Akt/ mechanistic target of rapamycin (mTOR) signaling pathways and are sensitive to PI3K and PI3K/mTOR inhibitors, suggesting another possible therapeutic approach for both near-haploid and low-hypodiploid B-ALL [6].

The PI3K/Akt/mTOR cascade is hyper-activated in several hematological malignancies [14] and up-regulation of this signaling pathway is related to poor prognosis and glucocorticoids (GC)-resistance [15]. Our group previously reported that NALM-16 near haploid cell line is sensitive to PI3K inhibition, improving GC therapeutic effects and/or overcoming GC-resistance [16].

In addition, selective p110δ inhibitors showed anti-leukemic effects in B-ALL cells and in a B-ALL mouse model [17]. In particular, a p110δ-specific inhibitor, GS-649443, was able to extend survival in a mouse xenograft model using near haploid cells and could also synergize with the conventional chemotherapeutic drug Cytarabine [18].

### 2.2. Hyperdiploidy

Hyperdiploidy is observed in approximately 25% of childhood B-ALL and is associated with a favorable outcome. Ras pathway alterations and epigenetic modifications are frequent events in high-hyperdiploid ALL [8], as well as CEBPE, ARID5B, PIP4K2A, and BMI1 genetic mutations [19,20,21], that are present in 50% of hyperdiploid patients [8]. Moreover, specific mutations of KRAS that lead to downstream phosphorylation of extracellular signal-regulated kinase (ERK) were found in hyperdiploid patients [8], suggesting that Ras/MEK/ERK network inhibition could be a good therapeutic target in high-hyperdiploid childhood B-ALL. Anyway, these observations warrant further investigations.

Finally, recurrent germline GRB2-associated-binding protein (GAB) 2 mutations were identified in high-hyperdiploid patients and could be evaluated as a putative novel predisposition factor to B-ALL development [21]. Interestingly, GAB2 is an upstream activator of Ras/MEK/ERK and PI3K/Akt/mTOR signaling pathways, though to bind SHP2 and the p85 regulatory subunit of PI3K [22].

## 3. Ph-Like B-ALL

Philadelphia Chromosome-like (Ph-like) ALL is a frequent ALL subtype that comprises up to 15% of pediatric B-ALL and its incidence increases with age, reaching 25% of young adults and 20% of adults with B-ALL [23,24]. It is associated with a poor outcome and it has been recognized as a provisional entity in the 2016 revision to the WHO classification [5]. This B-ALL subtype has a gene expression profile similar to Ph-positive ALL but lacking the canonical *BCR-ABL1* fusion gene that arises from the reciprocal translocation between chromosomes 9 and 22 [25]. The BCR-ABL1 oncoprotein has a constitutively activated TK enzymatic activity which confers a proliferative and survival advantage by activating several downstream pathways, including Ras/MEK/ERK, PI3K/Akt/mTOR, and Janus kinase (JAK)/signal transducer and activator of transcription proteins (STAT) [26]. Likewise, Ph-like ALL harbors a kinase activated signaling that still results from a multitude of different genetic rearrangements and mutations.

Ph-like ALL was described almost 10 years ago by two independent studies that identified a subset of pediatric ALL characterized by a specific gene expression profile similar to that of Ph-positive ALL [27,28].

Den Boer et al., employed a gene expression-based approach to classify the major subtypes of ALL, in order to improve prognostic classification of ALL in children, and hierarchical clustering revealed B-ALL cases that, although lacking the BCR-ABL1 fusion protein, clustered together with Ph-positive ALL [27]. This novel ALL subtype, called Ph-like or BCR–ABL1-like ALL, was characterized by a high frequency of deletions in genes involved in B-cell development (e.g., *IKZF1*) and by an unfavorable prognosis, with disease free survival rates comparable to that of pediatric patients with the Ph-positive ALL [27]. Mullighan et al., deeply characterized copy number alterations in high-risk ALL, and reported the strong association between deletions or mutations of *IKZF1* and poor outcome [28]. In addition, by employing an independent gene expression signature, they defined *IKZF1-*mutated *BCR-ABL1*-negative cases with a gene expression profile as similar to *BCR-ABL1*-positive ALL [28].

Ph-like cases lack single founding genomic alterations, but are defined by multiple genetic aberrations that converge on TK and cytokine receptor signaling pathways [29]. These alterations can be classified into discrete subgroups, based on the signaling pathway they affect. These include Cytokine Receptor Like Factor 2 (*CRLF2*) rearrangements (*IGH-CRLF2* and *P2RY8-CRLF2*) or mutations (p.Phe232Cys), fusions involving ABL-class genes (*ABL1, ABL2, CSF1R, LYN, PDGFRA*, and *PDGFRB*), alterations activating JAK/STAT signaling (*IL7R, SH2B3, JAK1, JAK3, TYK2*, and *IL2RB* mutations/deletions, and *JAK2, EPOR* or *TYK2* rearrangements), Ras signaling pathways (*NRAS, KRAS*, and *PTPN11*) and other less common fusions (*FLT3, FGFR1, NTRK3* and *PTK2B*) [23,24,30].

About half of pediatric Ph-like ALL show alterations in *CRLF2* gene, a member of the type I cytokine receptor family [24,30]. CRLF2 is a receptor for thymic stromal lymphopoietin (TSLP) that forms a heterodimeric complex with interleukin 7 (IL7) receptor (IL7R) and is involved in controlling cell proliferation and development via STAT3, STAT5, and JAK2 pathways during normal B-cell development. In Ph-like ALL, rearrangements with the immunoglobulin heavy chain locus (*IGH-CRLF2*) and a focal interstitial deletion, joining *CRLF2* to the adjacent *P2RY8* gene (*P2RY8-CRLF2*), result in *CRLF2* overexpression and increase CRLF2 protein expression [24,30]. In addition, less common point mutations lead to CRLF2 homodimerization and constitutive kinase signaling [31]. However, *CRLF2* over-expression is not an independent poor prognostic indicator in children with B-ALL [32]. Aberrant CRLF2 expression very frequently co-occurs with JAK1/JAK2 activating mutations or other mutations deregulating JAK/STAT signaling (e.g., *IL7R* mutations) [24,30], and these genetic lesions collaborate to induce ligand-independent activation of downstream signal transduction pathways. Deregulation of JAK/STAT signaling may also be due to *JAK2* or *EPOR* rearrangements, or to additional alterations activating other JAK/STAT signaling genes (*IL7R, FLT3, SH2B3, JAK1, JAK3, TYK2,* and *IL2RB*), accounting for 8%, 4% and 12% of pediatric Ph-like ALL, respectively [24,30].

The second major subgroup of Ph-like ALL includes ABL class fusions, involving *ABL1, ABL2, CSF1R*, and *PDGFRB*, identified in about 12–14% of pediatric Ph-like ALL, whereas mutations involving the Ras pathway were identified in about 6% of cases [24,30].

Collectively, a deeper analysis of the genomic landscape of Ph-like ALL showed that only a minority of patients does not harbor kinase activating alterations, highlighting the potential of JAK and ABL inhibition in the treatment of these higher-risk patients.

Since kinase-activating alterations were identified in over 90% of pediatric Ph-like ALL [24], and most of them converge on clinically actionable signaling, there is a great interest in identifying Ph-like patients, aiming to improve their outcome by using TK inhibitors (TKi) and JAK inhibitors. Different preclinical studies supported the evaluation of TKI Imatinib or Dasatinib in combination with chemotherapy [33] and several case reports corroborate the efficacy of TKIs in refractory Ph-like ALL patients with *PDGFRB* fusions [34,35,36,37]. For that reason, Dasatinib is currently tested in combination with chemotherapy to improve the cure rate of pediatric patients (NCT03117751; NCT01406756). Concerning Ph-like ALL with JAK/STAT pathway lesions, the addition of Ruxolitinib (a JAK inhibitor) to combination chemotherapy to treat pediatric patients is currently tested in three clinical trials (NCT03117751; NCT02723994; NCT02420717).

Actually, it is important to remind the remarkable progresses achieved in the treatment of Ph-positive ALL after the development of ABL TKi, whose introduction dramatically improved the prognosis, with disease-free survival in pediatric patients exceeding 70% [3]. However, identification of Ph-like ALL is currently challenging, and appropriate assays are not yet available as ordinary diagnostic approaches [38].

## 4. iAMP21 B-ALL

Intrachromosomal amplification of chromosome 21 (iAMP21) B-ALL is characterized by amplification of a portion of chromosome 21. It occurs in about 2% of children with B-ALL and it is associated with an intermediate prognosis [39]. This cytogenetic abnormality is caused by intrachromosomal amplification on chromosome 21, with the gain of more than two or three extra copies of RUNX1 with loss of subtelomeric region [39,40]. *RB1* and *EBF1* deletions, CRLF2 activating rearrangements, gain of the X chromosome and partial deletion of chromosome 7 have been frequently found in iAMP21 B-ALL [41]. Moreover, recurrent somatic mutations of the Ras/MEK/ERK pathway have been identified [42] and the MEK1/2 inhibitor Selumetinib showed strong cytotoxicity to leukemic iAMP21-positive cells, offering a possible targeted therapeutic strategy for iAMP21 B-ALL [42]. Of note, Selumetinib is currently undergoing clinical trials for the treatment of adult and pediatric ALL (NCT03705507).

## 5. MEF2D-Rearranged B-ALL

Myocyte enhancer factor 2D (MEF2D)-rearranged ALL arise in around 4% of pediatric ALL and may be considered as a biologically distinct form of leukemia. MEF2D-rearranged ALL patients have a poor prognosis; thus, a novel therapeutic strategy should be considered for this B-ALL subtype.

The MEF2D gene encodes a transcription factor involved in the muscular and neuronal differentiation that is regulated by histone deacetylases (HDAC) [43,44,45]. MEF2D plays a critical role in early B-cell differentiation, regulating the transition from the immature B-cell stage to the subsequent phases of B-cell development, through a Ras/MEK/ERK-dependent mechanism [46].

Several fusion partners in MEF2D rearrangements have been identified so far, including Bcl9, CSF1R, DAZAP1, FOXJ2, HNRNPUL1, HNRNPH1, and SS18 [47,48]. All these rearrangements lead to expression of chimeric fusion oncoproteins, characterized by increased MEF2D transcriptional activity that causes hematopoietic cell transformation [47].

Bcl9, the most common fusion partner, is a co-transcriptional activator that forms a multiprotein complex required for transcription of Wnt/β-catenin-dependent genes. The role of Bcl9 in cell growth, survival and development of leukemia is well established [49]. Notably, Wnt/β-catenin pathway is involved in self-renewal of healthy stem cells [50], as well as of leukemic stem cells (LSCs) [51]. An aberrant increase in the levels of β-catenin exerts oncogenic effects via the activation of downstream gene expression programs. Therefore, inhibition of Wnt/β-catenin pathway is a novel promising therapeutic strategy for hematological malignancies. Of note, PRI-724, a CBP/β-catenin transcription inhibitor, has now entered early-phase clinical trials for hematological malignancies, where it displays a tolerable toxicity profile (NCT01606579, NCT02195440).

Even though Bcl9 is involved in Wnt/β-catenin signaling network, it is not yet clear if the MEF2D-Bcl9 fusion protein directly interferes with Wnt/β-catenin pathway [47]. Interestingly, the other MEF2D partner, SS18, is involved in Wnt/β-catenin signaling pathway [52]. In synovial sarcoma, it has been reported that SS18 and β-catenin may both induce Wnt-dependent genes transcription, although they use different molecular complexes resulting in a synergistic effect [53]. Moreover, SS18 high levels lead to Wnt/β-catenin axis activation in HEK293 cells [54].

Finally, expression of MEF2D-Bcl9 fusion in a B-ALL cell line induces cell growth, resistance to dexamethasone and increase of HDAC9 expression [47,48,55].

A preclinical study shows that MEF2D-rearranged primary cells are sensitive to panobinostat, a HDAC inhibitor (HDACi), probably through the inhibition of HDAC9, which is overexpressed in MEF2D-rearranged B-ALL, offering a possible therapeutic option for MEF2D-rearranged ALL [47].

Several HDACi have already undergone clinical trials for ALL, including Panobinostat, alone (NCT00723203) or in combination with conventional chemotherapeutic drugs in childhood patients (NCT01321346, NCT02518750), Entinostat (NCT01383447), or Vorinostat, combined with Bortezomib for infants (NCT02553460) or children (NCT01312818).

## 6. ZNF384-Rearranged B-ALL

ZNF384-rearrangements are observed in 5% of pediatric B-ALL patients and are related to an intermediate prognosis. The *ZNF384* gene encodes a C2H2 zinc finger transcription factor that regulates the promotors of different matrix metalloproteinases [56].

The ZNF384 rearrangement may involve several partners, including transcriptional regulators or chromatin modifiers, such as ARIDIB, BMP2K, CLTC, CREBBP, EWSR1, NIPBL, SMARCA2, SYNRG, TAF15, and TCF3 [57].

ZNF384-rearranged B-ALL show a characteristic immunophenotype, with low CD10 expression and expression of myeloid markers CD13 and/or CD33, while clinical features depend on the functional defect of the different fusion partner genes.

TCF3-ZNF384-rearranged B-ALL represents the most frequent fusion gene involving ZNF384 and is characterized by a poorer chemotherapeutic response and a higher frequency of relapse compared to the other ZNF384 rearrangements [58]. Recurrent mutations in RAS signaling pathway genes (*NRAS*, *KRAS*, *PIK3CD*, and *PTPN11*) have been identified in TCF3-ZNF384-rearranged B-ALL [59], and this may explain at least partly the aggressive clinical course of this B-ALL subtype.

In 2015, Ohara’s group isolated a novel recurrent histone acetyltransferase p300 (EP300)-ZNF384 gene fusion, found in about 1.5% of pediatric B-ALL and showing clinical features significantly different from the other ZNF384-positive patients. For instance, EP300-ZNF384-positive patients have a more favorable prognosis [60]. EP300 acts on chromatin remodeling and has an onco-suppressor activity [61]. Moreover, the EP300-ZNF384 samples show an up-regulation of JAK/STAT pathway and reduced DNA repair capacity [62].

EP300- and CREBBP-ZNF384 rearranged B-ALL result in loss of histone lysine acetyltransferase activity, reduction of histone acetylation and increased sensitivity to HDACi, opening the possibility to use these inhibitors for these specific B-ALL subtypes.

## 7. DUX4-Rearranged B-ALL

A recently discovered distinct B-ALL subtype is characterized by recurrent *DUX4* rearrangements and a peculiar gene expression profile.

Genetic alterations of *DUX4* account for about 4–7% of B-ALL in pediatric cases and increase to more than 15% in adolescent and young adults (AYA) B-ALL [63,64,65].

The presence of *DUX4*-rearrangements has a favorable impact on prognosis and is associated with a longer disease-free survival, irrespective of the presence of *IKZF1* alterations, that are well-known to confer poor prognosis in other B-ALL subtypes [64,66].

The *DUX4* gene is located in the subtelomeric region of chromosome 4q known as D4Z4. This region is highly polymorphic and, in normal genomes, it consists of repeated segments varying from 11 to more than 100, each containing a copy of the *DUX4* gene. The *DUX4* gene is normally exclusively expressed in germ cells during early stem cell development, but little is known about the function of the protein [67].

*DUX4* rearrangements to either *IGH* or, less commonly, *ERG* genes are somatic events [63,64,65]. *IGH-DUX4* and *ERG-DUX4* fusions result from insertions of a partial copy of *DUX4* into the *IGH* locus or into intron 3 of *ERG*, respectively, that relocate *DUX4* under the control of the partner gene enhancer, thus leading to aberrant overexpression of *DUX4.* Even though these fusions do not give rise to chimeric proteins, they result in a 3′ truncated DUX4 transcript, leading to a protein that retains both homeobox domains, consequently preserving its DNA-binding capacity [65].

In cell-based assays, DUX4 fusions exhibited oncogenic activity; by contrast, knockdown of the *DUX4* fusion transcript suppressed cell proliferation. Furthermore, in vivo DUX4-IGH fusion showed leukemogenic potential in transplantation assay in mice and the pro-B cells expressing DUX4-IGH gave rise to pro-B cell leukemia [64].

The associated gene expression pattern is similar to those previously described in a subtype of B-ALL enriched for *ERG* deletions and associated with a favorable prognosis [68]. Consistently, the majority of *DUX4*-rearranged ALL harbor *ERG* focal deletions [63,65], but ERG deregulation occurs in all *DUX4*-rearranged ALL through multiple mechanisms, including expression of aberrant *ERG* transcripts, intron retention and expression of *ERGalt*, an alternative transcript that utilizes a non-canonical first exon. In addition, DUX4 induces *ERGalt* expression, by binding to the alternative transcription initiation site of ERG in intron 6, that is the non-canonical first exon of *ERGalt*. In turn, *ERGalt* inhibits the function of wild-type ERG [65].

In *DUX4*-rearranged B-ALL, additional recurrent genomic alterations affect the lymphoid transcription factor genes *IKZF1* and *PAX5*. Mutations were detected also in transcription factors and transcriptional regulators including *MYC*, *MYCBP2*, *MGA* and *ZEB2*, in genes involved in activation of Ras signaling, cell cycle regulation, and epigenetic modifiers including *KMT2D*, *SETD2*, *ARID2* and *NCOR1* [63,65].

DUX4 could be a potential therapeutic target. Since aberrant expression of DUX4 was identified as a major factor in the etiology of facioscapulohumeral dystrophy (FSHD), several therapeutic approaches were developed to inhibit its expression in this context and could be potentially useful to treat *DUX4*-rearranged ALL. However, at present there are no clinical trials testing DUX4 inhibitors or preclinical evidence proving their efficacy in B-ALL harboring *DUX4* rearrangements. In addition, the complexity of the genomic region where DUX4 is located makes difficult the identification of these rearrangements without high throughput screening.

## 8. Pax5-Driven B-ALL

Alterations of the B-lymphoid transcription factor gene PAX5 represent the most frequent somatic mutation in childhood B-ALL patients. In B-ALL, PAX5 can be altered by different somatic genomic abnormalities, such as deletions, gene fusions, and point mutations [69]. PAX5 alterations are initiating, subtype-defining events in B-ALL, interfering with the normal B-lymphoid development and interacting with activated kinase signaling [47,70].

PAX5 is a transcription factor required for B-lineage development and maintenance [71]. It acts by activating the expression of B-lineage-specific genes, including *CD79a*, *CD19*, *CD21*, *BLNK*, *CD72* or by inhibiting the transcription of other inappropriate genes, such as *NOTCH1*, *PD1*, and *M-CSFR*. Two different subtypes of B-ALL show PAX5 alterations: PAX5alt and PAX5 P80R.

### 8.1. PAX5alt

The first subtype PAX5alt, or “PAX5 altered”, comprises all PAX5 rearrangements, sequence mutations and structural variants [47,72]. PAX5 rearrangements may occur with different partners, such as *POM121*, *BRD1*, *DACH1*, *HIPK1* and *JAK2* (PAX5-JAK2 rearrangement has been found in Ph-like ALL, leading to aberrant activation of JAK/STAT signaling pathway) and all these rearrangements are observed in approximately 11% of B-ALL childhood patients [73]. PAX5alt subtype is associated with an intermediate prognosis [47].

Further frequent alterations have been found in PAX5alt patients and involve different genes, including *CDKN2A*, *IKZF1*, *ETV6* and *LEF1*, or lead to epigenetic modifications in *KDM6A*, *KMT2A* or *ATRX* [70].

Through induced *PAX5* loss, Liu et al., reported that PAX5 deficiency contributes to leukemogenesis, by supporting B-ALL self-renewal and by blocking a differentiation program that can be re-engaged despite the presence of additional oncogenic lesions to B-ALL maintenance [74]. Importantly, PAX5 deficiency plays a fundamental role for both B-ALL pathogenesis and maintenance, through constitutively STAT5 activation [73,74,75].

It has been suggested that PAX5 activity needs a threshold under which it cannot induce the B-lineage differentiation program [71]. In this context, it would be important for B-ALL PAX5alt patients to develop therapeutic compounds aimed at restoring or mimicking full PAX5 activity.

Notably, *PAX5* loss increases c-myc expression and proliferation in B-ALL, opening the possibility to consider c-myc as a novel, potentially highly effective target against childhood B-ALL overexpressing c-myc and harboring PAX5 rearrangements and mutations.

Indeed, recent findings reported that bromodomains (BRD) extra terminal (BET) inhibitors (BETi), which act inhibiting *C-MYC* transcription, are strongly cytotoxic in primary B-ALL cell lines and xenografts [76] and in pediatric primary B-ALL samples [77]. Moreover, it has been reported that *PAX5* loss is accompanied by mutations in additional signaling pathways genes, including Ras and JAK/STAT [78], and BETi also act by inhibiting STAT5-dependent gene expression [79]. Clinical studies with Ras and JAK/STAT inhibitors in Pax5alt B-ALL patients are therefore warranted.

### 8.2. PAX5 P80R

The second PAX5-driven subtype is characterized by the presence of a unique PAX5 mutation (P80R) that leads to loss of PAX5 activity [80]. This mutation has been observed in around 2% of B-ALL childhood patients [81].

The outcome of PAX5 P80R-mutated patients is favorable. Interestingly, patients carrying this mutation are characterized by frequent aberrant activation of signaling pathways involved in cell growth, such as Ras, JAK/STAT, and PI3K/Akt/mTOR, demonstrating a close relationship between PAX5 activity and signaling pathways [70]. *NRAS*, *PTPN11* and *IL7R* genes are most commonly mutated in the PAX5 P80R subtype. Moreover, PAX5 P80R patients frequently harbor deletion of *CDKN2A* gene, enrichment of *MYC* and *E2F* target genes and mutations in signal transduction factor genes, particularly in genes activated by mTOR and involved in Ras signaling pathway [47,80].

Recently, germline hypomorphic mutations in the PAX5 subtype have been related to B-ALL susceptibility, such as the recurrent germline PAX5 mutation (p.G183S), that has been identified in a familial ALL [82,83].

## 9. ETV6-RUNX1-Like B-ALL

t(12;21) is the most common translocation in pediatric B-ALL, with a frequency of 25% [25], that involves *ETV6* and *RUNX1*, two transcription factors both essential for normal hematopoiesis. Although *ETV6-RUNX1* does not represent an independent predictor of prognosis, when age and white blood cell count at the time of diagnosis are taken into account in multivariate analysis, its presence is associated with a good prognosis, with event-free survival rates of approximately 90% [84].

In 2016, Lilljebjörn et al., described the gene fusion landscape of pediatric B-ALL [63]. Through RNA-sequencing and gene expression profiling, they were able to classify 191/195 (98%) cases into distinct genetic subtypes. In particular, they identified in-frame gene fusions in the majority of cases (65%), whereas the remaining cases were classified as high-hyperdiploid, hypodiploid and Ph-like. In addition, they defined a novel B-ALL subtype associated with *ETV6-RUNX1*-like gene-expression pattern in 3% of the original cohort. The *ETV6-RUNX1*-like cases fall into B-other cases (defined as B-ALL without genetic aberrations at diagnosis, for which the leukemia driver events are still unknown), representing 14% of this B-ALL subgroup. *ETV6-RUNX1*-like cases harbored co-existing *ETV6* and *IKZF1* aberrations, including alternative in-frame *ETV6* gene fusions (*ETV6-PMEL*, *ETV6-BORCS5*, *ETV6-NID1*), *ETV6* deletions (both intragenic or whole-gene deletions), out-of-frame *ETV6* fusions in combination with *IKZF1* in-frame (reciprocal *SETD5-IKZF1* and *IKZF1-SETD5*) or out-of-frame fusions, as well as *IKZF1* whole-gene deletions, thus suggesting that combined *ETV6* and *IKZF1* lesions may activate similar transcriptional programs, as the ETV6-RUNX1 fusion protein. Of note, both *IKZF1* and *RUNX1* encode transcription factors important for B-cell maturation.

A further study reported the association of *ETV6-RUNX1*-like ALL with CD27^pos^/CD44^low-neg^ immunophenotype [85]. The CD27^pos^/CD44^low-neg^ expression pattern was previously reported as distinct and exclusive of *ETV6-RUNX1*-positive ALL and these leukemic cells correspond to the physiological counterparts of less mature stage of B-cell precursors. In contrast, CD27^neg^/CD44^pos^ cells correspond to most of the *ETV6-RUNX1*-negative ALL and, in physiological conditions, appear later in B-cell precursor development [86]. Because a close genotype-phenotype correlation is exceptionally rare, Zaliova et al., deeply investigated several CD27^pos^/CD44^low-neg^ *ETV6-RUNX1*-negative B-other ALL cases [85]. Gene expression profiling revealed a significant biological similarity of these *ETV6-RUNX1*-negative B-other ALL cases to *ETV6-RUNX1*-positive ALL, since 5 out of 7 cases clustered within the *ETV6-RUNX1*-positive subtype and, therefore, were classified as *ETV6-RUNX1*-like ALL. Furthermore, single nucleotide polymorphism (SNP) array analysis, whole exome (WES) and whole transcriptome (RNAseq) sequencing defined their genomic background. All the five cases harbored *ETV6* genetic aberration, including partial monoallelic deletions and in-frame *ETV6-BORCS5* fusion. Co-occurring *ETV6* alterations were also identified, including loss of *ARPP21* (3/5), *PAX5* (2/5), *ATP10A* (2/5) and *BTG1* (2/5) genes and *IKZF1* aberrations (deletion, nonsense mutation, and gene fusion).

*ETV6-RUNX1*-like B-ALL is a clinically relevant pediatric B-ALL subtype with prognostic relevance, and its identification is essential to improve risk stratification of B-other ALL cases. The frequency of *ETV6-RUNX1*-like B-ALL reaches 5% [87] and in a large-scale international study *ETV6-RUNX1*-like B-ALL, likewise *ETV6-RUNX1*-positive subtype, was identified only in pediatric cases and it was associated with low risk [81].

## 10. NUTM1 Rearranged B-ALL

NUT Midline Carcinoma Family Member 1 (NUTM1) fusion represents a rare recurrent childhood rearrangement, defining an infrequent B-ALL subgroup (1–2%) with a favorable outcome that has been found exclusively in children [88]. NUTM1 acts as a chromatin modifier by recruiting EP300 to increase histone acetylation [89] and it is not normally expressed in leukemic lymphoblasts [88]. The NUTM1 rearrangement may involve different partners, such as ACIN1, BRD9, CUX1, IKZF1, SLC12A6, and ZNF618, all resulting in NUTM1 over-expression [47,81]. NUTM1 rearrangements characterize a rare and aggressive subtype of squamous cell carcinoma defined NUT carcinoma (NC), that predominantly affects teens and young adults [90]. In NC, NUTM1 recruits histone acetyltransferases and other transcriptional co-factors activating pro-proliferative and anti-differentiation genes, such as *MYC* [91].

It is well-established that BETi have strong antitumor activity in NC [92]. BETi are drugs that competitively block the binding of BET proteins (e.g., c-myc) to acetylated lysines of histones. Importantly, a BETi is currently undergoing clinical trials for the treatment of NC carcinoma (NCT01587703). Thus, the efficacy of BETi in NC treatment opens the possibility to extent the administration of these drugs to NUTM1 B-ALL patients to generate a unique therapeutic opportunity for this B-ALL subtype.

## 11. Targetable Signaling Pathways in B-ALL

**Figure 2:**
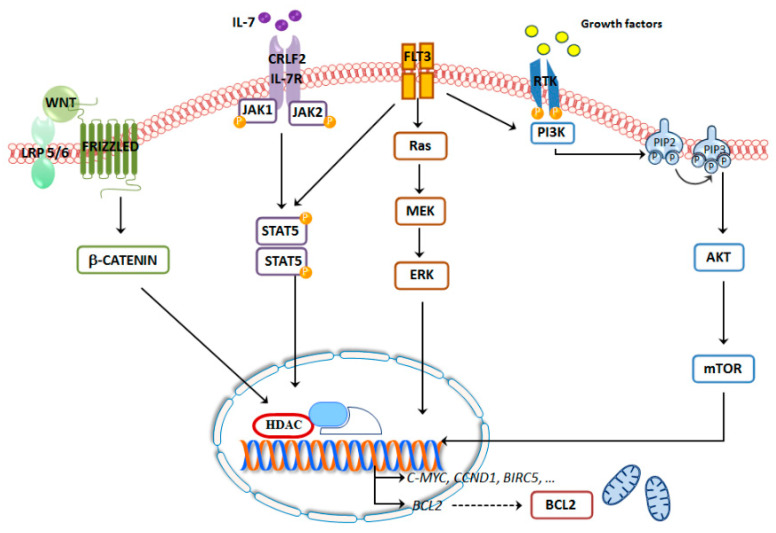
Most common signaling pathways involved in B-ALL.

The identification of novel B-ALL subtypes and their genomic anomalies provides a better therapeutic combination, with the aim to target several key survival pathways that will further ameliorate the therapeutic options, especially for refractory and relapsed pediatric patients, leading to spare to the younger patients the side effects elicited by conventional chemotherapeutic protocols (Figure 2, Table 1 and Table 2).

### 11.1. Ras

Activating mutations in Ras proteins are among the most common mutations in cancer. In B-ALL, the great majority of RAS mutations (98%) occur in *NRAS*, *KRAS*, *FLT3* and *PTPN11* genes. All of them play important roles in regulating cellular processes involved in tumorigenesis, including cell growth, survival, differentiation, and cell cycle regulation [93].

Recently, it has been showed for the first time that mutations in RAS-related genes are significantly more frequent in childhood B-ALL patients, reaching 44% of cases [94,95].

RAS mutations have been found in several B-ALL subtypes, including TCF3-ZNF384-rearranged B-ALL [59], hypodiploid [6], high hyperdiploid [8], Pax5alt [78] and P80R [70], TCF3-HLF-rearranged B-ALL [96], Ph-like [24] and iAMP21 B-ALL [42].

Notably, RAS mutations involving *NRAS, KRAS, FLT3* and *PTPN11* genes confer a bad prognosis [94,97,98,99], probably due to chemo-resistance mechanisms. Indeed, it has been previously reported that RAS mutations are related to chemo-resistance mechanisms [100]. RAS-mutated cells are resistant to Prednisolone, as well as to Vincristine and, more importantly, GC chemo-resistance can be eradicated by Ras pathway inhibition [101]. Ras proteins act mainly through two different signaling cascades, the Ras/MEK/ERK and the PI3K/Akt/mTOR pathways [93], that are often aberrantly activated in B-ALL.

### 11.2. PI3K/Akt/mTOR Signaling

PI3K/Akt/mTOR signaling network represents a critical signal transductioncascadeinvolved in several cellular functions, including mRNA translation, proliferation, survival, metabolism, and autophagy [102].

Dysregulation of PI3K/Akt/mTOR pathway is frequently reported in B-ALL [103] and correlates with worse prognosis and chemo-resistance in pediatric B-ALL patients [15].

Several inhibitors of the PI3K/Akt/mTOR axis were developed and tested in ALL patients [104]; nevertheless, only few of those drugs have been evaluated in childhood ALL patients [105].

However, drugs targeting PI3K/Akt/mTOR pathway showed promising results in preclinical models of B-ALL, through the direct inhibition of tumor cell growth and by reversal of GC resistance, although with a limited success when used as single anticancer agents [106]. On the contrary, these drugs demonstrated significant cytotoxic effects when combined with other chemotherapeutic agents, including Dexamethasone, Doxorubicin, L-Asparaginase, Methotrexate and Etoposide, and several progressed to clinical trials [106]. Encouraging results have been shown for relapsed childhood B-ALL [107], as also reported by a recent phase I clinical trial combining Everolimus (an allosteric mTOR inhibitor) with conventional chemotherapeutic drugs (NCT01523977).

The promising results obtained by the treatment of resistant and relapsed patients can be explained by the fact that up-regulation of PI3K/Akt/mTOR pathway is involved in chemo-resistance mechanisms, and specific inhibitors were able to overcome the resistance mechanisms, first of all by inhibiting the aberrant activation of the Ras/MEK/ERK pathway or the RTK overexpression [108].

The presence of *CRLF2* rearrangements, the most common alteration in Ph-like ALL, associates with aberrant JAK/STAT and PI3K/Akt/mTOR signaling pathways. In vitro studies demonstrated the interconnection between these two pathways, since stimulation with TSLP, which is the CRLF2 ligand, induced phosphorylation of both JAK/STAT and PI3K/Akt/mTOR pathway components, while treatment with the JAK inhibitor Ruxolitinib inhibited the activation of STAT5, ERK1/2 (downstream JAK/STAT signaling), Akt, S6RP, 4EBP1, and eIF4E (downstream PI3K/Akt/mTOR signaling), in both cell lines and primary samples from ALL patients [109]. Therefore, several studies investigated the therapeutic relevance of JAK/STAT and PI3K/Akt/mTOR inhibition. The mTOR inhibitor Rapamycin was evaluated in NSG xenograft models of specific subtypes of primary human B-ALL, demonstrating higher efficacy in Ph-like ALL than any other B-ALL subset, but particularly in B-ALL with altered CRLF2 and JAK/STAT signaling, in which it significantly prolonged patients’ survival [110]. Moreover, the combination of JAK2 and mTOR inhibitors produced robust anti-leukemic effects in Ph-like cell lines in vitro and in PDX cells cultured ex vivo, as well as in in vivo Ph-like B-ALL PDX models [111]. Therefore, these findings confirm that PI3K/Akt/mTOR pathway is a relevant target, at least in some cases of Ph-like ALL. Despite the therapeutic potential of mTOR inhibition in these subtypes, that might have relevant clinical implications, it is essential to identify which subsets of B-ALL are more likely to respond, in order to successfully test mTOR inhibitors in future clinical trials.

### 11.3. Ras/MEK/ERK Signaling

Constitutive activation of Ras/MEK/ERK signaling pathway is a frequent event in leukemic cells and may be caused by mutations of *RAS* genes. The Ras/MEK/ERK network plays a pivotal role in mediating the oncogenic effects of Ras, suggesting the potential efficacy of targeting this pathway in RAS-mutated cancers [112].

Among the Ras/MEK/ERK inhibitors, Trametinib, a highly specific and potent MEK1/2 inhibitor, is currently investigated in B-ALL. Interestingly, B-ALL RAS-mutant cells are sensitive to Trametinib, that in turn can re-sensitize resistant cells to Prednisolone [101]. Moreover, Trametinib and Prednisolone showed synergistic effects in pediatric relapsed B-ALL patients [95]. It follows that the relationship between RAS mutations and GC resistance may offer a novel promising alternative therapeutic strategy for childhood B-ALL relapsed patients [101,113,114].

### 11.4. Wnt/β-Catenin Signaling Pathway

Canonical Wnt/β-catenin pathway represents a crucial signaling axis involved in several cellular processes, including embryonic development, cell growth, wound healing, differentiation, apoptosis and migration [49]. This network acts through β-catenin that, translocating to the nucleus, activates several downstream target genes, such as *C-MYC*, *CCND1*, and *BIRC5* [115].

Wnt/β-catenin axis regulates hematopoiesis, through hematopoietic stem cells (HSCs) maintenance and self-renewal [116]. Aberrant activation of this signaling cascade is therefore associated to HSCs uncontrolled self-renewal, possibly generating LSCs and leading to hematologic neoplastic disorders [51]. Importantly, β-catenin inhibition blocks the self-renewal of drug-resistant LSCs, pushing these cells towards symmetric differentiation, without interfering with normal HSCs asymmetric differentiation [117,118].

The involvement of Wnt/β-catenin pathway in B-ALL development has been firstly showed by Khan et al., that reported that activation of Wnt proteins increases proliferation of B-ALL cell lines [119]. Then, Petropoulos et al., showed in mice that overexpression of a constitutive active Lymphoid Enhancer Binding Factor (LEF) 1 mutant is sufficient to develop B-ALL [120], thus identifying LEF1 as a new potential oncogene. Moreover, given that overexpressed LEF1 has been found in 25% of all B-ALL patients, it could represent an independent adverse prognostic factor [121].

In B-ALL, constitutive active Wnt/β-catenin pathway supports LSCs survival, together with bone marrow stromal cells [122], and in pediatric B-ALL is associated with drug-resistance [123,124]. Inhibition of Wnt/β-catenin pathway may therefore overcome drug resistance, by sensitizing B-ALL cells to Cytarabine treatment both in vitro and in vivo [122].

The potential of combining Wnt/β-catenin inhibitors with classical chemotherapy in B-ALL has been studied using ICG-001, a CBP/β-catenin transcription inhibitor. ICG-001 induced the reduction of self-renewal capacity of B-ALL cells, through downregulation of survivin (the product of *BIRC5* gene), thus overcoming drug-resistance in primary leukemia cells [124].

Moreover, recent findings reported that down-regulation of the β-catenin-dependent *BIRC5* gene induces in vitro chemo-sensitivity. Therefore, a phase I study combining the survivin mRNA antagonist EZN-3042 with re-induction chemotherapeutic drugs has been developed for pediatric relapsed B-ALL patients (NCT01186328) [125].

Wnt/β-catenin pathway dysregulation has been reported in different B-ALL subtypes, such as TFC3/PBX1 B-ALL, where Wnt16b is aberrantly activated by the expression of the E2A-Pbx1 fusion protein to induce leukemia [126,127]. Mazieres et al., confirmed that Wnt16b, β-catenin, Dvl2 and TCF4 are upregulated in TFC3/PBX1-positive cells and, more importantly, inhibition of Wnt16b leads to apoptosis [128].

Wnt/β-catenin pathway is also dysregulated in Ph-like B-ALL, where the hyper-activation of this signaling cascade may be triggered by epigenetic alterations, such as hyper-methylation of promoters of the Wnt/β-catenin antagonists SFRP, WIF1, and Dkk3 [129].

Finally, a crosstalk and correlation between up-regulation of Wnt/β-catenin and Ras/MEK/ERK signaling pathways has been demonstrated in different cancers, including relapsed childhood B-ALL [130,131], and it should require further preclinical studies [132].

### 11.5. Bcl2

The anti-apoptotic Bcl2 family proteins are overexpressed in many hematological malignancies, including B-ALL, where it has been found in more than 66% patients compared to healthy donors [133]. Indeed, in B-ALL, the balance of Bcl2 family proteins is often disrupted, pushing leukemic cells toward survival, and promoting leukemogenesis. That is why therapies targeting this anti-apoptotic regulator are considered as an attractive strategy for leukemia treatment.

A dual inhibitor of Bcl2 and Bcl-XL, named Navitoclax (ABT-263), resulted to be cytotoxic in Bcl2-dependent neoplasia, but it induced excessive cell toxicity during clinical trials, resulting in thrombocytopenia, and thus limiting its clinical application [134].

ABT-737, another Bcl2 inhibitor that neutralizes also Bcl-X, showed potent anti-leukemic activity in pre-clinical models, but its therapeutic use was limited because of lack of oral bioavailability and the induction of a strong decrease of circulating platelets, as reported for Navitoclax, so that these inhibitors never entered clinical phase III trials [135].

On the contrary, the highly selective Bcl2 inhibitor Venetoclax presented remarkable cytotoxic effects with significant reduced toxicity in comparison to dual Bcl2/Bcl-X inhibitors [136] in haematological malignancies, including acute myeloid leukemia (AML) [137,138] and non-Hodgkin lymphoma (NHL) [139]. In line with these data, studies of MLL-ALL cells in vitro [140] and in vivo [138] confirmed the anti-leukemic activity of Venetoclax as monotherapy for the treatment of hematological malignancies. Moreover, this Bcl2 inhibitor showed strong cytotoxic effects in B-ALL cell lines and in pediatric B-ALL PDX models [96,141].

Given its safe profile, Venetoclax has recently been approved by FDA for CLL patients with the 17p deletion [142]. Importantly, it also entered in a phase II clinical trials as monotherapy in patients with refractory and relapsed AML [143] and T-ALL [144].

Two clinical trials are analyzing the efficacy of Venetoclax in ALL. Firstly, safety and preliminary efficacy of Venetoclax combined with chemotherapy is being evaluated in a phase I clinical trial for pediatric ALL patients (NCT03236857) [145]. Another phase I trial will study the safety and pharmacokinetics of Venetoclax, Navitoclax, and chemotherapy in relapsed ALL (NCT03181126).

Recently, it has been reported that targeting Bcl2 is not effective as monotherapy for the treatment of childhood leukemia, but it becomes effective when associated with other drugs, such as inhibitors of survival pathways, thus allowing to induce apoptosis and to reduce side effects. Indeed, the anti-leukemic activity of Venetoclax treatment in B-ALL xenografts is weak, but it significantly increases when combined with Vincristine and Dexamethasone [141].

Given that Bcl2 activation could be the result of the dysregulation of activated signaling pathways, combined treatments with Bcl2 inhibitors and drugs targeting specific signaling pathways have been tested. For instance, targeting Bcl2 and PI3K/Akt/mTOR resulted in high cytotoxicity in vitro [107]. Moreover, combined treatment exerted synergistic growth-inhibitory effects and induced apoptosis in B-ALL cell lines and primary cells obtained from patients with resistant ALL patients [146]. The synergistic anti-leukemic effects of Bcl2 and PI3K/Akt/mTOR inhibitors may be explained by the fact that Bcl2 inhibitors may act by inducing apoptosis after mTOR inhibition, thus regulating both anti-apoptotic proteins, such as BCL-XL, and pro-apoptotic proteins, such as BAD or PUMA, as well as activators (BIM) or effectors (BAX) [107].

Further preclinical research supported these findings, highlighting that co-administration of PI3K/Akt/mTOR inhibitors and Venetoclax is synergistic in AML cells and in mice [147].

Promising results have been reported in TCF3-PBX1 B-ALL [127] and in hypodiploid B-ALL [6,11], suggesting that Bcl2 inhibition could be a encouraging strategy also for the treatment of these B-ALL subtypes. In conclusion, all preclinical data on Bcl2 inhibition in ALL confirmed the therapeutic potential of Bcl2 inhibition for B-ALL patients. Moreover, the FDA approval of Venetoclax for the treatment of CLL encourages its application also for pediatric B-ALL.

### 11.6. BET Proteins

The BET proteins are epigenetic readers that interact with acetylated histones and play important roles in proliferation and cell-cycle regulation [148]. In particular, they act as scaffolds for the activation of transcription factors and chromatin organizers required for the transcription of several pro-survival and anti-apoptotic genes, such as *MYC* and *BCL2*. Dysregulation of BET proteins is linked to the development of different diseases, including cancer [148]. That is why inhibition of the BET-histone interaction has recently gained increasing interest for the treatment of cancers characterized by altered histone acetylation and altered gene transcription, such as c-myc-dependent hematological malignancies and hematological malignancies with epigenetic alterations [149].

Several novel BETi have been developed by industrial and academic research groups, providing a novel pharmacological option for the treatment of cancer [150].

BETi showed an encouraging therapeutic potential in leukemia preclinical models, both as single agents and combined to other drugs. For instance, BETi showed cytotoxic effects in different hematological malignancies, including AML, lymphoma and ALL [76,151]. A strong anti-leukemic activity has also been reported in MLL-fusion leukemia, through the down-regulation of fundamental pro-survival genes, such as *BCL2*, *CDK6* and *MYC* [152].

In B-ALL, BET inhibition causes strong cytotoxic effects, down-regulates the expression of the oncogenes c-myc and IL7R and leads to apoptosis to B-ALL cell lines and primary patient-derived cells [76,77]. Moreover, BETi treatment of B-ALL PDX mouse models decreased the tumor burden and increased overall survival.

In addition, c-myc inhibition reduced LSCs in mice, confirming the involvement of c-myc in LSCs maintenance in ALL [153,154]. Interestingly, BETi deplete LSCs, acting through inhibition of c-myc expression. Several BETi are currently being evaluated in clinical trials for the treatment of hematological malignancies, including ALL, with promising responses (NCT02158858, NCT01943851, NCT01713582), [149,155].

Recently, possible combination of BETi with targeting pathways drugs have been evaluated, especially to overcome BETi resistance. Wnt/β-catenin signaling was shown as a driver of BETi resistance, restoring c-myc expression in AML. To confirm this, it has been showed that combined treatment synergizes in resistant AML cells [156]. Another study, using MLL-AF9-transduced progenitor cells, established that BETi-resistant cells displayed marked up-regulation of the Wnt/β-catenin pathway, both in vitro and in vivo [157].

Another possible synergism using BETi for the treatment of B-ALL is through the down-regulation of Aurora kinase (Ak) B [158]. AkB is involved in centrosome function, mitotic spindle assembly and cytokinesis. Its overexpression is often reported in different cancers, where it correlates with higher malignancy, higher proliferation, and worse outcome [159]. In B-ALL, AkB is aberrantly activated by c-myc and is often overexpressed [158]. In addition, in mice xenografted with primary B-ALL cells, BETi treatment caused a strong inhibition of AkB [158].

Finally, BETi also act by inhibiting STAT5-dependent transcription of pro-apoptotic genes, through synergistic effects with TKi in leukemic cells [79], and inhibiting NF-κB pathway in drug-resistant leukemia [160]. All these preclinical evidence suggests that BETi may have clinical implications in B-ALL subtypes characterized by aberrant oncogenic transcription driven by c-myc, including NUTM1 and PAX5 mutations.

### 11.7. HDAC

HDACs regulate chromatin structure through the removal of acetyl residues of core histones, modulating gene expression [161]. In humans, there are 11 classical HDAC isoforms, grouped into four classes. Altered expression and mutations of genes that encode HDACs play a critical role in leukemogenesis. Therefore, HDACs represent promising therapeutic targets for leukemia treatment [161]. HDAC inhibitors (HDACi) have been approved for the treatment of cutaneous T-cell lymphoma and Panobinostat, a selective HDACi, received FDA-approval in 2015 for the treatment of multiple myeloma patients [162].

HDACi activity has been also tested in preclinical models of B-ALL, demonstrating strong pro-apoptotic effects [163]. Recently, selective HDACi (HDAC1 and HDAC2 inhibitors) have been tested in a panel of B-ALL cells, including pediatric B-ALL cells, and in xenografted human leukemia patient samples, showing promising results [164]. Selective inhibitors showed a comparable sensitivity, compared to non-selective HDACi, but they inhibited leukemic growth and activated apoptosis both in vitro and in vivo, allowing to lower the accumulation of dsDNA lesions frequently associated to non-specific HDACi. The strong cytotoxic effects of selective HDACi appears to be more marked in B-ALL, as the activity of these inhibitors is reduced in other B-derived malignancies [164]. Moreover, selective HDACi that avoid inhibition of HDAC3 seem to show clinical benefits [165].

Selective HDACi are already approved for a lymphoid malignancy, cutaneous T-cell lymphoma and are currently undergoing clinical investigation for ALL (NCT01132573, NCT00462605, NCT01321346).

### 11.8. JAK/STAT

As mentioned above, Ph-like ALL is characterized by a range of genomic alterations that converge on a limited number of signaling pathways, more frequently the JAK/STAT. Besides activating alterations, including mutations, deletions and gene rearrangements that affect *IL7R, SH2B3, JAK1, JAK2, JAK3, TYK2, IL2RB, EPOR* or *TYK2*, also *CRLF2*-overexpressing ALL show aberrancies on the JAK/STAT pathway [109].

Preclinical in vitro studies demonstrated the ability of several fusion transcripts, for instance PAX5–JAK2, to confer cytokine-independent proliferation in mouse IL-dependent Ba/F3 cells and in IL7-dependent Arf^−/−^ pre-B cells expressing a dominant negative isoform of Ikaros [24]. Of note, such cellular models, harboring PAX5-JAK2, as well as human leukemic cells harboring ATF7IP-JAK2 or IGH-EPOR, were sensitive to Ruxolitinib, a selective JAK1/2 inhibitor [24]. The in vivo efficacy of Ruxolitinib treatment has also been reported in JAK2-mutated/CRLF2-rearranged ALL xenografts [110]. Further preclinical in vivo investigations confirmed that Ruxolitinib is the most potent agent against the majority of JAK/STAT activating alterations, including the JAK-family fusions ATF7IP-JAK2, PAX5-JAK2 (apart from those involving TYK2), and the following mutations: IL7R p.IsoLeu241-242ThrCys, JAK1 p.Leu782Phe, JAK3 p.Val670Ala. In addition, the cytostatic effects of Ruxolitinib monotherapy were significantly enhanced to the addition of Dexamethasone, supporting the combination of multiple agents to eradicate leukemia [166]. Recently, four different rearrangements of *EPOR* gene were exclusively identified in Ph-like ALL [66]. These rearrangements juxtapose *EPOR* to enhancer regions in immunoglobulin and immunoglobulin-like genes, resulting in deregulated *EPOR* expression and promoting leukemogenic potential in vivo. Human leukemic cells with *EPOR* rearrangements enhanced JAK/STAT signaling and were sensitive to JAK/STAT inhibition. More importantly, in human xenografts, the JAK1/2 inhibitor Ruxolitinib exhibited potent synergism with conventional chemotherapeutic agents, including Dexamethasone, Vincristine, and Daunorubicin, thus offering an alternative therapy for this subset of Ph-like ALL.

Collectively, these studies provided the rationale for testing JAK inhibitor-based therapies in the clinics. Indeed, current several phase I–II clinical trials are now testing the efficacy of Ruxolitinib in combination with chemotherapy in pediatric patients with activation of JAK/STAT signaling, including de novo high-risk *CRLF2*-rearranged and/or JAK pathway-mutant ALL or Ph-like ALL (NCT02723994, NCT02420717, NCT03117751).

### 11.9. ABL1 Kinase

The second largest group of mutations in Ph-like ALL comprises ABL-class fusions (ABL1, ABL2, CSF1R, PDGFRB), that are predicted to respond to ABL1 inhibitors. The efficacy of such TKi has been evaluated in preclinical in vitro and in vivo models, and the ABL fusion genes RCSD1-ABL1, RSCD1-ABL2, SSBP2-CSF1R and ETV6-ABL1 were sensitive to a number of ABL1 inhibitors, including Imatinib, Dasatinib, Nilotinib and Ponatinib [24,166,167]. In addition, a multitude of reports involving pediatric patients indicate the benefit of an early introduction of ABL1 inhibition following the identification of ABL-class fusions as well as the application of TKi monotherapy to eradicate MRD [34,35,36,37,167]. Accordingly, several phase I-II clinical trials are testing dasatinib in combination with chemotherapy in patients with Ph-like ALL with ABL1-class fusion (NCT03117751, NCT02420717).

## 12. Conclusions

B‑ALL is the most prevalent cancer in childhood. Despite considerable improvements in overall survival, a proportion of pediatric B-ALL has still unsatisfactory outcomes with the current treatments.

The recent sequencing-based studies greatly improved the classification of childhood B-ALL, enabling the identification of novel subtypes characterized by druggable genomic anomalies. Since the genetic background impact on initiation, progression and outcome of this disease, these novel findings provide a better understanding of the pathobiology of B-ALL. In addition, these genetic abnormalities are clinically relevant for risk stratification, treatment choice and prognosis.

Although the outcome of several new B-ALL subtypes remains poor, targeted therapeutic strategies may potentially show a great impact on B-ALL prognosis, especially for subtypes that actually lack efficient therapeutic protocols.

Dysregulated signaling pathways are often interconnected in B-ALL, giving the opportunity to combine targeted compounds to enhance treatment efficacy and ultimately eradicate leukemia cells. In light of this, targeted therapy offers an unrivalled chance to simultaneously block multiple signaling while changing the course of cancer.

We expect in the next future great improvements in diagnostic tests, in order to support the routine identification of the novel molecular alterations that defines the new B-ALL subtypes, as well as continued investigations aiming at find new druggable targets that may enhance anti-leukemic efficacy while decreasing side effects. In conclusion, the recent efforts in basic, translational and clinical research might hopefully render B-ALL a curable disease.

## Figures and Tables

**Table 1:** Targetable pathways of childhood B-ALL subtypes.

B-ALL Subtype	Prognosis	Altered Signaling Pathways	Target	Ref
**MEF2D**	Unfavorable/Intermediate	Ras/MEK/ERK, Wnt/β-catenin	HDAC	[47]
**ZNF384**	Intermediate	Ras, JAK/STAT	HDAC	[56]
**DUX4**	Very Favorable	Ras		[63]
**Hypodiploidy**	Unfavorable/Intermediate	Ras, PI3K/Akt/mTOR	Bcl2	[6]
**Hyperdiploidy**	Very Favorable	Ras/MEK/ERK	MEK	[8]
**Pax5 alt**	Intermediate	c-myc	BET	[73]
**Pax5 P80R**	Favorable	Ras, c-myc, IL7		[80]
**Ph-like**	Unfavorable	JAK/STAT, ABL1, Ras, PI3K/Akt/mTOR/IL7	mTOR, JAK	[25]
**ETV6-RUNX1-like**	Favorable		ABL1	[25]
**NUTM1**	Very Favorable	c-myc	BET	[88]
**iAMP21**	Intermediate	Ras/MEK/ERK	MEK	[42]

**Table 2:** Clinical trials activated for hematological malignancies and pediatric ALL involving the most important signaling pathways (clinicaltrials.gov).

Drug	Target	Disease	Clinical Trials
**Everolimus**	mTOR	Pediatric ALL	NCT01523994
**Selumetinib**	MEK1/2	Pediatric ALL	NCT03705507
**PRI-724**	β-catenin	Hematogical malignancies	NCT01606579 NCT02195440
**Venetoclax**	Bcl2	Pediatric ALL	NCT03504644 NCT04029688 NCT03236857
**Molibresib**	BET proteins	Hematogical malignancies	NCT01943851
**OTX015**	BET proteins	Hematogical malignancies	NCT01713582
**Panobistat**	HDAC	Pediatric ALL	NCT01321346 NCT02518750
**Entinostat**	HDAC	B-ALL	NCT01383447
**Vorinostat**	HDAC	Pediatric ALL	NCT02553460 NCT01312818
**Ruxolinib**	JAK/STAT	Pediatric ALL	NCT02723994 NCT02420717 NCT03117751
**Dasatinib**	ABL1	Pediatric Ph-like ALL	NCT03117751 NCT02420717 NCT01406756
